# Coordination and Perceived Support for Return to Work: A Cross-Sectional Study among Patients in Swedish Healthcare

**DOI:** 10.3390/ijerph19074040

**Published:** 2022-03-29

**Authors:** Erik Berglund, Emilie Friberg, Monika Engblom, Åsa Andersén, Veronica Svärd

**Affiliations:** 1 Division of Insurance Medicine, Department of Clinical Neuroscience, Karolinska Institutet, SE-171 77 Stockholm, Sweden; emilie.friberg@ki.se (E.F.); monika.engblom@regionstockholm.se (M.E.); veronica.svard@ki.se (V.S.); 2 Department of Public Health and Caring Sciences, Uppsala University, SE-751 22 Uppsala, Sweden; asa.andersen@pubcare.uu.se; 3 Division of Social Work, Department of Social Sciences, Södertörn University, SE-141 89 Huddinge, Sweden; 4 Department of Social Work in Health, Karolinska University Hospital, SE-171 76 Stockholm, Sweden

**Keywords:** sick leave, return to work (RTW), vocational rehabilitation, RTW coordination (RTWC), experienced quality in healthcare, support, emotion, healthcare utilization

## Abstract

Background: Receiving support from a return-to-work (RTW) coordinator (RTWC) may be beneficial for people on long-term sick leave. The aim of this study was to investigate whether the number of contacts with an RTWC and their involvement in designing rehabilitation plans for the patients were associated with perceiving support for RTW, emotional response to the RTWC, and healthcare utilization. Methods: In this cross-sectional study, 274 patients who had recently been in contact with an RTWC in Swedish primary or psychiatric care answered questions regarding their interaction with an RTWC, perceived support for RTW, and emotional response to the RTWC. Results: Having more contact with an RTWC was associated with perceiving more support in the RTW process (adjusted OR 4.14, 95% CI 1.49–11.47). RTWC involvement in designing a rehabilitation plan for the patient was associated with perceiving more support in the RTW process from an RTWC and having a more positive emotional response to the RTWC. Conclusions: From the patient’s perspective, this study indicates that the involvement of an RTWC and receiving a rehabilitation plan that an RTWC has helped to design might be perceived as important in the RTW process.

## 1. Introduction

Sick leave is associated with considerable social and economic costs for both individuals and society, especially in the case of long sick leave [1]. Those on long-term sick leave often have a lengthy return-to-work (RTW) process, and workplace engagement, coordination, and cooperation between client, employer, and different stakeholders is stated to be crucial for a successful RTW process [2,3,4].

Several strategies and interventions for shortening sick leave and promoting RTW have been put forward. The scientific evidence favors multimodal interventions and those that involve the employer or workplace [5,6]. It has been suggested that the RTW process comprises several phases: the beginning of sick leave, involvement in treatment, rehabilitation with health professionals, gradual RTW, and post-RTW sustainability [7,8]. In all, a number of phases, environments, systems, interventions, and interactions spread across multiple stakeholders may be involved. The involvement of multiple stakeholders after long-term sick leave implies that the RTW outcome is influenced by the collaboration and coordination between them. For a more successful RTW process, RTW coordination is often recommended for improving the collaboration and coordination between different stakeholders and the person on sick leave [9,10]. 

In most countries, the RTW coordinators (RTWCs) are situated in workplaces or insurance companies [11]. In Sweden, RTWCs are most often referred to as rehabilitation coordinators and have been introduced over the last decade, usually in public healthcare settings. Since 2020, the healthcare system in Sweden is obliged to offer patients on sick leave rehabilitation coordination, if needed [12]. The main purpose of an RTWC is to mediate and facilitate RTW for people on sick leave by coordinating and giving individualized support [13]. The RTWC may initiate and collaborate with different healthcare professions such as physicians, psychologists, and physiotherapists. The RTWC also collaborates with stakeholders outside healthcare, such as employers, the Social Insurance Agency, and the social services. The RTWC may also be involved in creating a plan for the RTW process [14].

Although RTWCs are considered to play an important role in creating and maintaining a working alliance between stakeholders in the RTW process [10], research findings are not unanimous about their effect on RTW outcomes. Some studies demonstrate that RTWC interventions have a positive effect on RTW [5,15,16]; others have found no or adverse effects [17,18].

Most previous studies on RTWC have explored whether they have an effect on RTW and the length of sick leave. Little research has, however, been carried out into how patients perceive the coordination of their RTW process in a public healthcare setting. It is essential that the patients themselves appreciate and find the coordination meaningful and helpful. Otherwise, there is a risk that patients find it pointless or do not use the coordination intervention at all. The patients’ perspective is, therefore, important for optimizing the coordination efforts. Previous studies of the patient perspective on coordination are based on interviews with small numbers of participants [19,20,21,22,23]. These studies have found that RTWCs are perceived to enhance communication and collaboration between stakeholders and the transfer of knowledge between them. They are also seen as providing patients with daily structure, support, and encouragement. Hitherto, no quantitative studies have been carried out of the associations between variations in received RTWC interventions and patients’ self-reported experiences, focusing on benefit from the patient’s perspective. There is a need for additional knowledge about how patients experience the support from RTWCs. Considering the patients’ point of view for evaluating self-rated treatment outcomes is immanent to the achievement of outcomes that are relevant to patients in general healthcare [24], as well as in rehabilitation [25].

The aim of this study was to investigate whether the number of contacts with an RTWC or having a rehabilitation plan that an RTWC had helped to design were perceived as supportive for RTW and associated with a positive emotional response to the RTWC. A secondary aim was to explore whether the number of contacts with an RTWC or having a rehabilitation plan that an RTWC had helped to design was associated with healthcare utilization.

## 2. Materials and Methods

### 2.1. Study Population and Procedure

This study was conducted as a cross-sectional study with patients who had recently been in contact with an RTWC in primary or psychiatric care in Region Stockholm, Sweden. To find such patients, RTWCs working at 44 primary healthcare centers and 12 psychiatric clinics were asked to distribute a questionnaire to all patients they had been in contact with in February and March 2020. The questionnaire and two reminders were sent out between June and August 2020. In total, the RTWCs identified 1086 eligible patients. The RTWCs were provided with material to send to their patients. This included the questionnaire, information about the study and voluntary participation, and a response envelope addressed to the project leader at the Karolinska Institutet, ensuring complete participant anonymity. Of the 292 responses to the questionnaire (27% response rate), 18 were excluded because they answered that they had not been in contact with an RTWC. A total of 274 patients were included in the study. Of these, 84.3% were patients from primary healthcare centers and 15.7% from psychiatric clinics.

### 2.2. The Questionnaire

A questionnaire was developed consisting of 50 questions plus partial questions. It included questions about demographics, how the patient had come into contact with an RTWC, experience of that RTWC contact, and diseases. The questionnaire was pilot tested in a reference group consisting of representatives from different patient organizations, and some revisions were made based on their comments. The particular variables used in the analyses are described in the following sections.

### 2.3. Explanatory Variables

Interventions received from an RTWC. The number of contacts with an RTWC was operationalized as the number of sessions that a patient had had with an RTWC, face-to-face or by phone. Information about whether the RTWC had helped to design a rehabilitation plan for the patient was assessed by the question: “Did the rehabilitation coordinator help you to create a plan for treatment/rehabilitation?” Response options were: “yes”, “no”, or “do not know”. Based on the first two options, a dichotomous variable was created for the binary logistic regression analyses.

Occupational status, demographics, and disease burden. Answers about occupational status were dichotomized into two categories: those who had an employment contract or were self-employed and those who did not. Demographic data used in this study were sex, age, educational level (categorized as compulsory or secondary school, and university), and country of birth (categorized as Sweden, rest of Europe, or rest of the world). Disease burden was based on a list of illnesses, diagnoses, and disabilities in the questionnaire and used as a cumulative measure of disease burden. Disease burden was dichotomized into one disease and two or more diseases for the binary logistic regression analyses.

The number of specific diseases listed by participants varied widely, and in this study, the four first diseases listed by participants were summarized aiming to explore the most common diseases.

### 2.4. Outcome Variables

Perceived support for RTW given by the RTWC was assessed by the statement about whether the RTWC had “Supported my opportunities to RTW”. Answers were collected with the following alternatives: (1) not at all, (2) to a small extent, (3) partially, and (4) highly. In this study, a cutoff was used to dichotomize support for RTW from an RTWC into more (score 3–4) and less (score 1–2) perceived support for the binary logistic regression analyses.

The emotional response to RTWC index was based on six indicators developed for this study. The following statements were used to measure emotional response to the RTWC intervention: “I felt…” (1) “strengthened in my situation”, (2) “more empowered”, (3) “more optimistic”, (4) “disappointed”, (5) “more sad or depressed”, and (6) “annoyed”. Answers were collected through a four-point Likert scale ranging from 1 = “not at all true” to 4 = “completely true”. The index was calculated so that a higher score indicated a more positive emotional response to the received RTWC intervention. The Cronbach’s alpha for the emotional response index was 0.92. For the binary logistic regression analyses, the index was dichotomized into more-positive and less-positive emotional responses, based on the median value in the study group (median value 3.5).

Healthcare utilization describes the use of health services by persons for the purpose of preventing and curing health problems, promoting maintenance of health and well-being, or obtaining information about one’s health status and prognosis [26]. The healthcare utilization variable in this study was assessed by the number of times a patient stated that he or she had visited healthcare professionals. Similar approaches are common when assessing healthcare utilization [27,28]. The following statements were used: “In the past six months I have visited the following: a doctor, medical specialist, nurse, health social worker, psychologist, physiotherapist, or occupational therapist.” For the binary logistic regression analyses, the sum of healthcare use was dichotomized into “higher healthcare utilization” and “lower healthcare utilization”, based on the median value in the study group (median value 11).

### 2.5. Analyses

The data were analyzed using chi-square tests to compare differences in distributions, ANOVA for differences between mean values, and Kruskal–Wallis H test for comparisons of ranked data. Binary logistic regression models were used to compute odds ratios (ORs) and 95% confident intervals (CIs) for associations between the number of contacts with an RTWC, having a rehabilitation plan that an RTWC had helped to design, occupational status, demographics, disease burden, perceived support for RTW, emotional response to the RTWC, and healthcare utilization. Both crude and adjusted logistic regression analyses were carried out. The adjusted models included number of contacts with an RTWC, having a rehabilitation plan that an RTWC had helped to design, occupational status, demographics, and disease burden. All tests were two-sided, and a level of *p* < 0.05 was considered statistically significant. The statistical analyses were performed using SPSS statistics (IBM Corp, Armonk, NY, USA), version 27.0.

## 3. Results

The study population consisted of 79.6% women and 18.6% men. The average age of the study group was 47.0 years (SD 10.4). See Table 1. Most participants (77.4%) were employed or self-employed (women 81.0% and men 66.0%). In total, 62.1% reported two or more diseases, and this was more common among women (64.9%). When summarizing the first four diseases listed by the participants, the following were most common: burnout/fatigue symptoms (24.8%); other mental disorders or neuropsychiatric disorders (37.2%); pain disorders (8.6%); other primarily somatic disorders, injuries, disabilities, or symptoms (29.4%).

### 3.1. Received Interventions from a Rehabilitation Coordinator

The majority (72.3%) of the patients in the study sample had been referred to the RTWC by another healthcare professional. Furthermore, 14.2% had been contacted by the RTWC directly, 5.8% had sought out an RTWC themselves, while 7.7% stated that they had made contact another way. On average, 6.7 (SD = 4.2) contacts with an RTWC were reported, and 79.5% stated that an RTWC had been involved in designing their rehabilitation plan. No statistically significant differences were found between women and men (Table 1).

### 3.2. Perceived Support for RTW and Emotional Response to RTWC

In the total study population, 22.9% of patients perceived less support for RTW from the RTWC, and 77.1% perceived a higher support for RTW from the RTWC. The emotional response to RTWC index was on average 3.3 (SD = 0.7) and the median value was 3.5. No statistically significant differences were observed between women and men (Table 1). 

A Kruskal–Wallis H test showed that there was a statistically significant difference in number of contacts with an RTWC between the different response categories in perceived support for RTW from RTWC, *H* (3) = 20.9, *p* = 0.001 (Table 2).

Table 2 presents the results of a chi-square test that showed statistically significant difference between those who had and those who not had a rehabilitation plan that the RTWC had helped to design and perceived support for RTW from RTWC (*p* = 0.001).

### 3.3. Logistic Regression Models

Table 3 presents the odds ratios of number of contacts with an RTWC, having a rehabilitation plan that an RTWC had helped to design, occupational status, demographics, disease burden, perceived support for RTW from an RTWC, emotional response to an RTWC, and higher healthcare utilization.

In the adjusted logistic regression model, there was a statistically significant association between number of contacts with an RTWC and perceiving more support for RTW from an RTWC (adjusted OR 4.14, 95% CI 1.49–11.47). There was also a statistically significant association between having a rehabilitation plan that an RTWC had helped to design and perceiving more support for RTW from an RTWC (adjusted OR 7.99, 95% CI 2.84–22.54). No statistically significant associations were found between occupational status and support for RTW from an RTWC or between demographics or disease burden and support for RTW from an RTWC. In the adjusted model, 29.5% of the variance in perceived support for RTW from an RTWC was explained (Table 3).

In the adjusted logistic regression model, there was no statistically significant association between number of contacts with an RTWC and emotional response to the RTWC. There was, however, a statistically significant association between having a rehabilitation plan that the RTWC had helped to design and reporting a more positive emotional response to the RTWC (adjusted OR 7.98, 95% CI 2.98–21.39). In the adjusted logistic regression model, 22.7% of the variance in the emotional response to RTWC index was explained (Table 3).

There was no statistically significant association between number of contacts with a RTWC or having a rehabilitation plan that the RTWC had helped to design and healthcare utilization. The logistic regression analysis showed that having more than one disease was statistically significantly associated with more healthcare utilization (adjusted OR 1.85, 95% CI 1.02–3.34). In the adjusted logistic regression model, 6.3% of the variance in healthcare utilization was explained (Table 3).

## 4. Discussion

The results showed that a higher number of contacts with an RTWC was associated with reporting that the RTWC was perceived as being more RTW supportive. Having a rehabilitation plan that the RTWC had helped to design was associated with higher perceived support for RTW and a more positive emotional response to the RTWC. These results suggest that people on sick leave may benefit from a greater number of contacts with an RTWC during the RTW process, especially if the intervention includes RTWC involvement in designing rehabilitation plans.

Previous research has found that perceived adequacy of professional support may influence RTW [29]. RTWCs were implemented to improve the quality of the interaction between persons on sick leave, the healthcare services, and other stakeholders in order to improve the success of the RTW process [23]. This study cannot judge the quality of the interactions, but it does indicate that a higher number of contacts with an RTWC may be perceived as more supportive of RTW and positive emotional response to the RTWC. This indicates that more interactions with an RTWC in the RTW process may be beneficial for patients.

The Swedish Association of Local Authorities and Regions has recently found that 76% of the responding RTWCs in their national sample helped the patients with designing rehabilitation plans [30]. This is in line with our findings, showing that 79.5% of the patients stated that they had a rehabilitation plan that an RTWC had helped to design. Our study especially explored RTWC involvement in designing rehabilitation plans and the associated benefits for patients. RTWC involvement in designing rehabilitation plans was found to be associated with them being regarded by patients as more supportive for RTW. RTWC involvement in designing rehabilitation plans was also associated with a more positive emotional response to the RTWC. These findings indicate that an RTWC’s intervention may be enhanced by their involvement in developing rehabilitation plans to support RTW. This seems to be appreciated by the patients and may help to provide a clear and structured plan for RTW. The results are in line with previous studies [14,31], which found that developing RTW plans was an important component of RTWCs’ work, together with face-to-face contact, ergonomic worksite evaluation, communication/coordination between stakeholders, and identifying barriers to and facilitators of RTW. This study assessed the patient’s perspective of RTWC involvement in these plans. However, patients can have a rehabilitation plan from their employer as well as from the general healthcare services, and the questionnaire did not ask respondents which of these they were referring to. The format and specific content of rehabilitation plans can certainly vary between patients, and this study has not looked at the content and quality of these plans or how well they were followed up.

RTWCs can have a wide variety of tasks, such as applying laws and policies relating to work absence and RTW, planning the RTW, and collaborating with employees and their employers during a patient’s sick leave [11,32]. In most settings, the role of an RTWC is to manage the RTW trajectory for a person on sick leave [14], which implies that the RTWC should be in contact with a patient over a certain period of time. Previous research suggests that interventions involving an RTWC may be more time consuming than other approaches [10,14]. It has also been suggested that there may be some time-lag effect with RTWC interventions [33], which indicates that the benefits of an RTWC intervention do not materialize rapidly. Providing additional RTWC support in the late phase of transitioning back into the workforce has also been found to be beneficial for the RTW process [33]. Although the present study did not measure the duration of the RTWC intervention or how far into the RTW process the patients answered the questions, the results indicate that a higher number of contacts with an RTWC was associated with perceiving the RTWC to be more supportive of RTW. From an implementation and adherence standpoint, it is important that RTWCs can meet the needs of the patients to become a requested and active resource in healthcare. For the purposes of providing support that patients experience as advantageous, the results in this study highlight several aspects related to the intervention that RTWCs provide. Such as the importance of RTWCs being able to facilitate a greater number of contacts and being involved in designing the rehabilitation plans.

This study was conducted from a patient perspective and with self-reported data. It cannot answer whether the RTWC involvement really had an effect on RTW and sick-leave duration. This study was carried out during the COVID-19 pandemic, when many healthcare contacts had to shift from usual face-to-face meetings to other forms of contact, such as telephone. However, previous research has identified both face-to-face contacts with an RTWC [14] and telephone contacts (during follow up) with an RTWC [33] as important, and patients have themselves reported telephone contacts with an RTWC to be beneficial and supportive [23]. In this study, the total number of RTWC contacts included both face-to-face meetings and telephone contacts. The implications of this for the results are unclear. 

Even if a substantial degree of variance was explained by the outcome measures regarding perceived RTWC support for RTW and the emotional response to the RTWC, there was still much unexplained variance. This indicates that there are factors other than the RTWC intervention that influence outcomes.

The secondary aim of this study was to explore whether variations in the RTWC interventions were associated with healthcare utilization. The results did not indicate such an association. This finding is in line with a previous randomized controlled trial of an RTWC intervention, which did not find any effect on healthcare utilization [34]. There is a need to further investigate whether and how RTWC interventions may influence healthcare utilization. In the short term, an RTWC may increase the use of healthcare by initiating contacts with a variety of healthcare professionals who are needed for a patient’s RTW. In the long run, however, there may be a reduction in healthcare utilization if patients with RTWCs receive adequate rehabilitation and, thus, achieve a rapid RTW. In addition, RTWCs may also, sometimes, contribute to limit the number of healthcare contacts, and a previous qualitative study found that RTWCs sometimes advise patients to limit the number of healthcare contacts—indicating that RTWCs also may reduce healthcare utilization in the short term [23].

Another reflection to make based on our results, is that 79.6% of the respondents were women, which is a notably higher proportion considering that 62% of the total people on sick leave in Sweden were women the year the study took place [35]. This might mirror the fact that RTWCs in primary healthcare centers have a particular focus on people on sick leave due to mental disorders, in combination with the fact that nearly half of the sick leave cases for women are due to mental disorders, compared to nearly 40% among men.

To find patients with experience of a rehabilitation coordination, RTWCs were contacted by the researchers and asked to distribute the surveys. This resulted in an accurate study sample of patients with experience of an RTWC but created no opportunity for dropout analysis. However, as in all studies using questionnaire data, there is a risk of non-response bias. Patients who had a poor experience of an RTWC intervention may also be underrepresented in the study sample. Furthermore, we do not have information about the participants’ status in the RTW process. Some may have already returned to work when they answered the questionnaire, while others may have been in ongoing contact with their RTWC. All participants had had contact with an RTWC for at least three months, some may have had such contact for more than six months.

Since both explanatory and outcome variables were self-reported, there is a risk of recall bias. With a cross-sectional design, it is not possible to determine the temporal or cause–effect relationship between the RTWC intervention and the outcomes, which is a limitation. It is, however, reasonable to assume that the RTWC intervention may affect outcomes related to the perceived support from the RTWC, rather than the other way around. Nevertheless, whether the direction of causality in the underlying assumption is appropriate is a matter for discussion. For example, patients with a poor RTWC experience may have avoided (further) contact with an assigned RTWC. On the other hand, patients with a better experience may have asked for, attended, and remembered more contacts, thereby also reporting a higher number in the questionnaire. There is a need to address the research question of whether RTWC interventions predict support for RTW in randomized intervention studies and longitudinal studies. 

This study used binary logistic regressions for the primary analyses, which is appropriate for analyzing survey data in cross-sectional research designs [36]. However, the odds ratio measures can overestimate the prevalence ratio in some cases [37].

There was no information about non-responders, meaning that we do not know whether certain groups of patients are underrepresented in the data. We do know, however, that the response rate was lower among psychiatric patients (17%) than among primary healthcare patients (31%). This is in line with the literature: patients with mental disorders are often among the more “hard-to-reach” patient populations [38]. It is possible that some important confounders, such as job type, were not included in the multivariate analysis to assess the adjusted association between the explanatory measures and the outcomes. There is a need to further investigate how people perceive their rehabilitation coordination and whether RTWC involvement is more beneficial in some patient groups than in others.

## 5. Conclusions

This study of patients who had recently been in contact with an RTWC in primary or psychiatric healthcare found that a higher number of contacts with an RTWC was associated with reporting more support from an RTWC for RTW. Having a rehabilitation plan that an RTWC had been involved in designing was associated with both reporting more support for RTW from the RTWC and a more positive emotional response to the RTWC intervention.

The main findings imply that it might be possible to improve how patients perceive support for RTW from an RTWC by offering a greater number of contacts and by more RTWC involvement in designing their rehabilitation plans.

## Figures and Tables

**Table 1:** Characteristics of study participants.

		Women *n* = 218 (79.6)	Men *n* = 51 (18.6)	Total *n* = 274 (100)
Recruiting center or clinic	Primary healthcare centers	84.9	80.4	84.0
	Psychiatric clinics	15.1	19.6	16.0
Age, years	Mean (SD)	46.6 (10.5)	48.4 (9.8)	47.0 (10.4)
Education level	Compulsory or secondary school	35.5	50.0	38.2
	University	64.5	50.0	61.8
Country of birth	Sweden	77.9	84.3	79.1
	Rest of Europe	10.6	11.8	10.8
	Rest of world	11.5	3.9	10.1
Occupational status	Employment contract or self-employed	81.0 *	66.0 *	77.4
	Not in paid work	20.0 *	34.0 *	22.6
Disease burden	One disease	35.1	50.0	37.9
	Two or more diseases	64.9	50.0	62.1
Number of contacts with RTWC ^a^	Sessions with RTWC, median (Md), mean (SD)	6, 6.7 (4.0)	5, 6.7 (5.0)	6, 6.7 (4.2)
Having a rehabilitation plan that an RTWC had helped to design	No	19.9	23.3	20.6
	Yes	80.1	76.7	79.5
Perceived support for RTW from RTWC	Md, mean (SD) ^b^	3.0, 3.1 (1.0)	3.0, 3.2 (1.1)	3.0, 3.1 (1.0)
	Perceiving less support ^c^	23.3	21.1	22.9
	Perceiving more support ^c^	76.7	78.9	77.1
Index of emotional response to RTWC	Md, mean (SD) ^d^	3.5, 3.4 (0.7)	3.3, 3.2 (0.9)	3.5, 3.3 (0.7)
	≤3.5 ^e^	52.9	62.5	54.7
	>3.5 ^e^	47.1	37.5	45.3
Healthcare utilization	Md, mean (SD)	11, 13.5 (10.1)	11, 13.3 (10.6)	11, 13.1 (10.2)
	Visited healthcare service 11 times or less in the last six months ^f^	51.4	60.0	53.0
	Visited the healthcare service 12 times or more in the last six months ^f^	48.6	40.0	47.0

Characteristics of study participants distributed based on gender (1.8% did not answer or stated “other”). Figures as percentages if not stated otherwise. ^a^ Return-to-work coordinator (RTWC). ^b^ Perceived support for return to work (RTW) from RTWC ranging from 1 (= not at all) to 4 (= highly). ^c^ Experience of RTWC support for RTW was dichotomized into: less support (score 1–2) and more support (score 3–4). ^d^ The emotional response to RTWC index ranging from 0 (= not at all true) and 4 (= completely true). ^e^ The emotional response to RTWC index was dichotomized based on the median value. ^f^ Healthcare utilization was dichotomized based on the median value. * *p* < 0.05.

**Table 2:** Emotional support for return-to-work, number of contacts with RTWC, rehabilitation plan that the RTWC had helped to design, distributed on support for return-to-work from RTWC.

Perceived Support for Return to Work from RTWC	Highly *n* = 97 (45.8)	Partially *n* = 66 (31.1)	To a Small Extent *n* = 28 (13.2)	Not at All *n* = 21 (9.9)	*p*-Value
Index of emotional response to RTWC ^a^, median (Md), mean (SD)	4, 3.76 (0.30)	3, 3.31 (0.53)	3, 3.01 (0.62)	2, 2.25 (0.94)	0.01 ^b^
Healthcare utilization, Md, mean (SD)	11, 12.49 (9.35)	12, 15.95 (11.18)	12, 14.07 (11.44)	11, 15.29 (13.37)	0.34 ^b^
Number of contacts with RTWC, Md, mean (SD)	7, 8.00 (4.51)	6, 6.48 (3.74)	5, 5.18 (2.96)	4, 4.67 (2.87)	0.01 ^b^
Not having a rehabilitation plan that the RTWC had helped to design, *n* (%)	10 (10.3)	21 (33.9)	13 (48.1)	16 (76.2)	0.01 ^c^
Having a rehabilitation plan that the RTWC had helped to design, *n* (%)	87 (89.7)	41 (66.1)	14 (51.9)	5 (23.8)	

Return-to-work coordinator (RTWC). ^a^ The emotional response to RTWC index ranging from 0 (= not at all true) and 4 (= completely true). ^b^ Kruskal–Wallis H test. ^c^ Pearson chi-squared test.

**Table 3:** Binary logistic Regressions presenting odds ratios explaining perceived support for return to work from an RTWC, emotional response to RTWC, and reporting higher healthcare utilization.

	Perceived Support for Return to Work from RTWC		Emotional Response to RTWC		Healthcare Utilization	
	Crude OR (95% CI)	Adjusted Model OR (95% CI)	Crude OR (95% CI)	Adjusted Model OR (95% CI)	Crude OR (95% CI)	Adjusted Model OR (95% CI)
Number of contacts with an RTWC						
RTWC sessions ≤ 3	Ref.	Ref.	Ref.	Ref.	Ref.	Ref.
RTWC sessions > 3	3.81 ** (1.87–7.75)	4.14 ** (1.49–11.47)	2.23 * (1.20–4.15)	1.62 (0.71–3.69)	1.39 (0.79–2.46)	1.67 (0.80–3.48)
Having a rehabilitation plan that the RTWC had helped to design						
No	Ref.	Ref.	Ref.	Ref.	Ref.	Ref.
Yes	8.08 ** (3.50–18.68)	7.99 ** (2.84–22.54)	8.27 ** (3.31–20.64)	7.98 ** (2.98–21.39)	1.18 (0.61–2.26)	1.11 (0.53–2.33)
Occupation status						
Not in paid work	Ref.	Ref.	Ref.	Ref.	Ref.	Ref.
Employment contract or self-employed	1.65 (0.79–3.43)	2.41 (0.86–6.76)	2.27 * (1.21–4.28)	2.15 (0.99–4.66)	0.89 (0.51–1.58)	0.82 (0.41–1.65)
Sex						
Men	Ref.	Ref.	Ref.	Ref.	Ref.	Ref.
Women	0.88 (0.37–2.07)	0.65 (0.18–2.32)	1.48 (0.78–2.83)	1.27 (0.57–2.82)	1.42 (0.76–2.65)	1.35 (0.63–2.89)
Age	0.99 (0.96–1.03)	1.04 (1.00–1.09)	1.01 (0.98–1.03)	1.02 (0.99–1.05)	0.99 (0.97–1.02)	0.99 (0.96–1.02)
Education level						
Compulsory or secondary school	Ref.	Ref.	Ref.	Ref.	Ref.	Ref.
University	1.30 (0.67–2.53)	1.31 (0.52–3.32)	0.94 (0.57–1.56)	1.15 (0.59–2.24)	1.15 (0.70–1.88)	0.75 (0.41–1.38)
Country of birth						
Sweden	Ref.	Ref.	Ref.	Ref.	Ref.	Ref.
Rest of Europe	2.37 (0.67–8.34)	1.86 (0.39–8.93)	0.27 * (0.10–0.75)	0.27 (0.07–1.11)	0.87 (0.40–1.89)	0.98 (0.34–2.81)
Rest of world	1.58 (0.43–5.76)	1.39 (0.25–7.71)	1.08 (0.45–2.60)	1.72 (0.52–5.68)	0.73 (0.33–1.66)	0.79 (0.29–2.18)
Number of diseases						
One disease	Ref.	Ref.	Ref.	Ref.	Ref.	Ref.
Two or more diseases	0.76 (0.38–1.51)	0.86 (0.33–2.21)	0.79 (0.47–1.32)	0.82 (0.43–1.57)	2.04 ** (1.22–3.41)	1.85 * (1.02–3.34)
		29.5%		22.7%		6.3%

Odds ratio (OR), 95% CI: 95% confidence interval for experiencing more support for return to work (RTW) from a return-to-work coordinator (RTWC), emotional response to RTWC, and reporting higher healthcare utilization. * *p* < 0.05, ** *p*< 0.01. Adjusted model = Number of contacts with an RTWC + having a rehabilitation plan that the RTWC had helped to design + occupation status + demographics + disease burden.

## Data Availability

The data are not publicly available due to ethical restrictions.
